# Editorial: Cognitive and emotional mechanisms of time perception

**DOI:** 10.3389/fpsyg.2022.1069460

**Published:** 2023-01-04

**Authors:** Philip A. Gable, Timothy P. McCoy

**Affiliations:** Department of Psychological and Brain Sciences, University of Delaware, Newark, DE, United States

**Keywords:** time perception, emotion, temporal distortion, motivation, arousal

Over the years, major contributions have been put forth to clarify the emotional and cognitive aspects of time perception. Articles in the special issue on Cognitive and Emotional Mechanisms of Time Perception consider this question through several different lenses. Embedded throughout the manuscripts are themes that cut across multiple lines of research. These themes include mechanisms of time perception, the role of action in time perception, individual differences influencing time perception, and task differences measuring temporal distortion. Below we highlight articles relating to these themes and how they expand our understanding of time perception.

## Mechanisms of time perception

One of the most common themes of the articles in the special issue is that many of the authors discuss mechanisms of how emotion influences time perception. Zhou et al. is a strong example of testing the potential mechanistic effects of arousal using both experimental manipulations of the mechanism and the observed mediational effects of the mechanism. Despite the rigorous mechanistic approach, the authors should be commended for noting an alternative view, that their results “could be interpreted as allowing individuals to prepare more for subsequent approaching behavior, rather than a byproduct of the increased arousal.” (p. 9).

Another potential mechanism of time perception is noted in the review paper by Gable et al., which focuses on the role of motivation as a mechanism influencing time perception. Their review highlights a growing body of research showing that approach motivation tends to speed the passing of time, but withdrawal motivation tends to slow the passing of time. Along these lines, the paper by Matsuda and Nittono measured self-reported approach and avoidance reactions to their experimental stimuli.

While these papers stand as examples of mechanistic investigations, few studies of time perception clearly define a mechanism and experimentally or statistically test whether the mechanism plays a role in temporal changes. There is a growing need for research examining mechanisms of emotion-time perception interactions.

## The role of action in time perception

Another common theme throughout this special issue is the role of action in time perception. For example, Ma et al. found that positive and negative faces caused greater CNV event-related potential amplitudes. Because the CNV is a neural measure of one's preparation to act, this would suggest that temporal estimates might be influenced by the motivation to act. These results also highlight the importance of applying physiological measures to study mechanisms of time perception. This theme is discussed by Matsuda and Nittono. Specifically, they highlight the benefit of using physiological measures like skin conductance when proposing arousal as a mechanism for alterations of time perception.

The role of action is suggested in two other articles (Jia et al.; Yue et al.). Jia et al. investigated temporal perception during immoral phrases. Participants were more likely to perceive immoral phrases as being displayed longer than neutral or disgusting phrases. The authors conclude that these results do not support arousal as a mechanism, but suggest that embodied reactions toward immoral phrases may cause the shift in time perception.

Yue et al. found that people tend to have a more positive bias toward the future self as opposed to the past self. It could be that people view themselves as moving toward a future self and away from a past self. However, He et al. raise the importance of cultural distinctions in views about time, noting that English speakers view time horizontally whereas Mandarin speakers view time vertically. This cultural perspective may influence whether individuals approach the future differently. As individuals move *forward* or *up* through time, they could feel like they are approaching a future self but withdrawing from a past self.

## Individual differences influencing time perception

As with many psychological constructs, individual differences influence our sense of time. Individual differences are evident in the paper by Ma et al. demonstrating that emotional awareness plays a role in the perception of time. Differences emerged in their findings when comparing those who were most emotionally aware with those who were least emotionally aware.

Work by Weng et al. finds that individual pain reactions played an important role in determining people's responses during a time bisection task. The authors also highlight the importance of studying individual differences in time sensitivity, revealing that time sensitivity during a time bisection task was the leading factor influenced by longer administrations of pain.

## Task differences measuring temporal distortion

The variety of tasks used to assess time perception may cause variance and inconsistencies across studies. For example, in this special issue, researchers used time estimation, time reproduction, and time bisection tasks. Sometimes tasks were retrospective, other times they were prospective. The time window investigated ranged from long time intervals (ranging from seconds to minutes) to short time intervals (in the range of hundreds of milliseconds). Indeed, Lin et al. highlight the unique properties of time distortion for sub- and supra-second durations of time perception, finding that color sensitivity differed between shorter vs. longer windows of time.

The papers in this special issue also highlight the variety of ways to measure time perception. For example, the paper by Droit-Volet and Gil was the first to introduce emotional faces to a novel temporal reproduction task. Notably, their results in the neutral (control) condition replicated prior results. This paper outlines the importance of future studies to replicate past effects when introducing new or derivative versions of time perception tasks.

## Conclusion

This brief editorial fails to capture the breadth and depth of each of the articles in this special issue. You are invited to explore the expanse of knowledge from a collection of time researchers inquiring into how we perceive time and what alters our sense of time. These investigations serve to propel our understanding of time perception forward. We are hopeful this work will lead other researchers to expand upon the cognitive and affective mechanisms of time perception.

## Author contributions

PG contributed as the lead author to the writing of this editorial. TM contributed as the co-author to the writing of this editorial. Both authors contributed to the article and approved the submitted version.

